# FIDES Athlete Development Programme: project background and study protocol of an embedded multiple case study

**DOI:** 10.1136/bmjsem-2024-001898

**Published:** 2024-02-08

**Authors:** Aron Laxdal, Monica Klungland Torstveit, Sofia Ryman Augustsson, Ådne Ausland, Daniel Bjärsholm, Jørgen Bagger Kjær, Mariah Larsson, Susanne Linner, Anna Melin, Felicia Radovan, Bård Erlend Solstad

**Affiliations:** 1 Department of Sport Science and Physical Education, University of Agder, Kristiansand, Norway; 2 Department of Sport Science, Linnaeus University, Kalmar, Sweden

**Keywords:** Adolescent, Sports, Young, Female

## Abstract

Most sports science research revolves around male subjects. As a result, most of the knowledge and practices within sports are male-centric. Failing to take the biological, psychological and social (biopsychosocial) particularities of females into account is believed to hinder optimal sports participation, development and performance, with potential negative effects on the health and well-being of females. To close the knowledge gap and alleviate these issues, we aim to develop and evaluate a 12-video educational intervention that addresses female-specific subject matter: the FIDES Athlete Development Programme. The study is designed as an embedded multiple case study where at least 1320 Swedish female athletes aged 13–16 will participate, in addition to their parents and their coaches. The girls will be recruited through their sports clubs, with half being exposed to the FIDES Athlete Development Programme and the other half serving as control cases. The primary outcomes are well-being and sporting experience. To further increase our understanding of the intervention and its implications, interviews and focus group interviews with a reference group of girls and focus group interviews with a randomly selected subsample of coaches and parents will also be performed. The project is approved by the Swedish ethics committee (number: 2023-05264-01) and will be carried out in compliance with the Declaration of Helsinki. Results from the project will be published open access in peer-reviewed journals, at national and international conferences, in mass media, and a PhD thesis. The anonymised data will be made openly available in a data repository.

**WHAT IS ALREADY KNOWN ON THIS TOPIC**

- Most sport-related knowledge is male-centric.
- This can have a detrimental effect on female athletes for various reasons.

**WHAT THIS STUDY ADDS**

- This study aims to develop and evaluate an intervention to enhance female-specific knowledge among athletes, coaches and parents.

**HOW THIS STUDY MIGHT AFFECT RESEARCH, PRACTICE OR POLICY**

- The long-term goal is to enhance the sporting experiences and improve the well-being of girls in organised sports.

## Introduction

 With increased gender equality worldwide and an acknowledgement of the health benefits of sports participation, the number of females who actively participate in exercise and sports is increasing rapidly.^1^ However, female athletes are often regarded as less able, less competitive and frailer than male athletes.^2^ Structural impediments are, therefore, in place that hinder females’ long-term participation, development and performance.^3^,^5^ One of these impediments is that most sports science research is performed on male subjects at various athletic levels.^6^ Consequently, the typical coaching education programmes are based on male-centred research.^7 8^ Despite many similarities between males and females, there are also fundamental differences between the sexes. Not taking those differences into account may lead to suboptimal development, body image misconceptions, negative peer dynamics, increased injury prevalence and premature drop-out for female athletes.^9^

Even though awareness of this issue exists within the sporting environment, more must be done to address it at the grassroots level.^10^ Many coaching methods are still being used, and the same stigmatising philosophies prevail, despite being outdated and sometimes even discriminating. A better approach would be to create a more enlightened sporting environment where girls and young women work with their bodies and not against them.^2 11^ As a result, there have been calls from various stakeholders for measures that improve the well-being and sporting experiences of girls and young females in organised sports.^12^ These calls are reasonable, seeing as the continued participation, development, health and performances of girls and young females are contingent on equitable and inclusive sporting environments that consider their biological, psychological and social (biopsychosocial) particularities.^13^

While the need for more relevant female-specific knowledge within the sporting environment is problematic, it stems at least partly from the suboptimal dissemination of relevant information from researchers and national sports organisations. In other words, the knowledge exists but is not being disseminated to the relevant parties. As a result, many coaches and parents do not possess the necessary knowledge to tackle or guide female athletes through the various biopsychosocial sporting challenges they encounter during puberty and adolescence.^9 14^ Parents and coaches who would be expected to guide the girls through this transition have been found to have a narrow and simplistic perception of puberty, body image, shape and weight.^15 16^ Thus, it is imperative that not only the girls but also parents and coaches become more informed about the developmental changes occurring during puberty and adolescence.

Adolescence is characterised by an increased sensitivity to social stimuli and a heightened need for peer interaction and acceptance. Adolescent female athletes have an increased likelihood of both risky and prosocial behaviour in the presence of their peers.^17^ Furthermore, it is also worth noting that a recent meta-analysis suggests stronger peer influence effects over shorter periods, which, in turn, reinforces the need to examine adolescent athletes’ perceptions of the ‘peer-created’ motivational climate.^18^ Future interventions are, therefore, encouraged to aid adolescent athletes in their continuing navigation of the social environment in organised sports settings, which is likely to reduce the risk of poor mental health in adolescence.

### The study’s aims and objectives

The main aims of the FIDES Athlete Development Programme (ADP)^1^ are twofold. First, to develop a cost-effective and sustainable educational intervention for girls in organised sport and to evaluate the FIDES ADP using the Reach, Effectiveness, Adoption, Implementation and Maintenance (RE-AIM) framework. The hope is that this intervention can increase the well-being and improve the sporting experiences of young female athletes in organised sports by disseminating female-specific knowledge to key stakeholders.

## Methods

### Study design

The study is designed as an embedded multiple case study.^19^ Case study designs are commonly used in evaluation research to describe the activities of a group by focusing on an in-depth exploration of the actual ‘case’.^19 20^ They are especially useful when doing interventions or evaluations on a contemporary issue within a real-life context.^19^

To the best of our knowledge, this is the first study that aims to develop, implement and evaluate an educational intervention to enhance the sporting experiences and improve the well-being of girls in organised sports. The study is, therefore, explorative in nature, which is appropriate when there is limited experience with or knowledge of using new methods to investigate a phenomenon.^19^ While adolescent girls are the main unit of analysis, the embedded design will also encompass the coaches and the parents, who are important stakeholders in the microenvironment.^21^

### Participants

The study will include girls aged 13–16 years participating in organised sports, their parents and their coaches. We will focus on six popular sports for girls in Sweden: football, handball, gymnastics, horseback riding, floorball and swimming. These sports provide a mix of team/individual sports, male/female/neutral dominant sports, Swedish/other ethnicity athletes, and leanness/non-leanness sports, which is important, seeing as different contexts are likely to lead to different results.^19 22^ The participants will be recruited strategically from two regional sports federations in Sweden (Småland and Skåne), with clubs serving as the recruitment unit.

### The intervention

The FIDES ADP will consist of 12 videos of 5–10 min focusing on challenges likely to affect girls in organised sports. Videos were chosen as a medium as they are easily consumed, scalable, accessible and effective.^23^ However, recent research has shown a need for the active involvement of end-users (ie, the target population) in cocreation and to attend to the production quality so that the educational intervention is as relevant, informed and effective as possible.^24^ Every video will, therefore, revolve around a subtheme deemed relevant to the target audience through a presurvey among young female athletes representing the target population. Before implementing the intervention, a feasibility study will be performed on a single team, where all 12 videos will be watched on a compressed timeline. The participants will answer questions related to the content and implementation of the FIDES ADP. This process is important in optimising the full-scale intervention’s implantation and assessing the study’s viability.

### Variables and analyses

Background information, main outcome variables, secondary outcome variables and moderators (see table 1) will be measured at baseline. Excluding background information and puberty status, the same variables will also be measured during the postintervention and post-post follow-up stages. In addition, a selection of evaluation questions will be asked regarding the content of the videos after each viewing.

**Table 1:** An overview of the measures that will be used to evaluate FIDES ADP

Measure	Scale
Main outcomes	
Well-being	The WHO-5 Well-Being Index^30^
Sporting experiences	Positive and Negative Experience^31^
Secondary outcomes	
Peer interactions	Peer Motivational Climate in Youth Sport Questionnaire^32^
Need for competence	Basic psychological need satisfaction^33^
Body image	The Body Appreciation Scale^34^
Psychological flexibility	Children’s Psychological Flexibility Questionnaire^35^
Perceived social support	The Parent-Initiated Motivational Climate in sport^36^
Knowledge gains	Questions to the girls, coaches and parents regarding content and learning outcomes after each video
Moderators	
Socioeconomic status	The Family Affluence Scale III^37^
Life satisfaction	A single-item measure of life satisfaction^38^
Puberty status	The Tanner scale^39^The LEAFQ^40^
Coach interpersonal behaviours	A Tripartite Measure of Interpersonal Behaviours of Coaches^41^

ADPAthlete Development ProgrammeLEAF-QThe Low Energy Availability in Females Questionnaire

Given that female athletes are nested within sports teams, which, in turn, are nested within sports clubs, it is important to analyse the effects of individuals being nested within teams and clubs, respectively. Successive groupings in hierarchical data may cause individuals within particular sports teams to share certain properties, including socialisation patterns, attitudes and goals.^25^ The FIDES ADP will use multivariate growth curve analysis for three-level data. A longitudinal multivariate mixture model will likely allow for a more nuanced understanding of heterogeneity among groups and individuals.^25 26^ A sample size calculation based on a longitudinal multivariate mixture model indicated that 50 girls from each sport discipline must complete the 6-month post-post follow-up survey. With an expected drop-out rate of 30% during the intervention period, 660 girls will need to be included in both the intervention and the control group, respectively.^27^

The study will not be achieved through a single method, so a series of interviews will be performed to understand the underlying phenomena at play better. A reference group of 12 girls aged 13–16 years (2 representatives from each sport) will be interviewed individually and in focus groups at various process stages. Focus groups of randomly sampled parents and coaches will also be interviewed before and after the intervention. Six triads of athletes, parents and coaches will undergo prospective focus group interviews during the intervention period to abide by the RE-AIM principles. Focus group interviews were chosen as they often promote discussion and vigorous responses from participants.^28^ The participants will be randomly allocated into different groups, aiming to take advantage of the group dynamics in an organised discussion where the group can share their views of a specific experience or topic.^28^ The focus group interviews will be semistructured in an appropriate setting, and the participants will receive the overall discussion topics when asked to participate in the study. This procedure is meant to allow them to prepare for the focus groups and ensure that there will be no surprises when the interviews take place. The qualitative data will be analysed using a reflexive thematic analysis.

### Patient and public involvement

To better address the concerns of adolescent girls in 2024, a presurvey of females aged 16–24 years who participate in organised sport will explore which topics young female athletes in Sweden feel they lack knowledge on and how they would like to consume the videos that will relay that knowledge. Additionally, the reference group will advise the research group throughout the project. Such cocreation has been found to affect study outcomes favourably in the past despite mostly being limited to the earlier stages of the project.^29^ The presurvey and the reference group will also address concerns regarding possible adverse effects.

### Dissemination

The project will be coordinated by the principal investigator, associate professor, AM, at the Linnaeus University in Sweden, and the study results will be disseminated as peer-reviewed articles, national and international conference presentations, popular science contributions, and in a PhD thesis, irrespective of the outcomes.

## Data Availability

No data are available.

## Footnotes

For the last three3 years (2021-–ongoing), several researchers (Associate Professor Bård Erlend Solstad, Associate Professor Aron Laxdal, and Professor Monica Klungland Torstveit) at the University of Agder (UiA; Kristiansand, Norway) have worked towards establishing a research centre focusing on females in exercise and sport. The research centre is named FIDES (Female Inclusion & Development in Exercise and Sport). However, the FIDES ADP is a research project financed by the Kamprad Family Foundation and will be developed, implemented, and evaluated by researchers at the Linnaeus University in Sweden. The principal investigator of the FIDES ADP is Associate Professor Anna Melin (anna.melin@lnu.sese).
